# Prognostic Prediction for Patients with Hepatocellular Carcinoma and Ascites: Role of Albumin-Bilirubin (ALBI) Grade and Easy (EZ)-ALBI Grade

**DOI:** 10.3390/cancers15030753

**Published:** 2023-01-25

**Authors:** Jia-I Liao, Shu-Yein Ho, Po-Hong Liu, Chia-Yang Hsu, Yi-Hsiang Huang, Chien-Wei Su, Ming-Chih Hou, Teh-Ia Huo

**Affiliations:** 1 Division of Gastroenterology, Department of Medicine, Taipei Veterans General Hospital, Taipei 112, Taiwan; 2 School of Medicine, National Yang Ming Chiao Tung University, Taipei 112, Taiwan; 3 Division of Gastroenterology and Hepatology, Min-Sheng General Hospital, Taoyuan 330, Taiwan; 4 Department of Internal Medicine, University of Texas Southwestern Medical Center, Dallas, TX 75390, USA; 5 Department of Medicine, Renown Regional Medical Center, Reno, NV 89502, USA; 6 Institute of Clinical Medicine, National Yang Ming Chiao Tung University, Taipei 112, Taiwan; 7 Department of Medical Research, Taipei Veterans General Hospital, Taipei 112, Taiwan; 8 Institute of Pharmacology, National Yang Ming Chiao Tung University, Taipei 112, Taiwan

**Keywords:** ALBI grade, EZ-ALBI grade, hepatocellular carcinoma, ascites, prognosis

## Abstract

**Simple Summary:**

Ascites is a hallmark of advanced cirrhosis and often coexists in patients with hepatocellular carcinoma (HCC). The albumin-bilirubin (ALBI) grade and EZ (easy)-ALBI grade are used to indicate the severity of liver dysfunction in HCC, but the predictive accuracy of these two models in HCC patients with ascites is unclear. We found that ascites is highly prevalent (22.5%) in HCC; higher ascites grade was associated with higher ALBI and EZ-ALBI scores and linked with decreased overall survival. In the Cox multivariate analysis, serum bilirubin level > 1.1 mg/dL, creatinine level ≥ 1.2 mg/dL, α-fetoprotein ≥ 20 ng/mL, total tumor volume > 100 cm^3^, vascular invasion, distant metastasis, poor performance status, ALBI grade 2 and 3, EZ-ALBI grade 2 and 3, and non-curative treatments were independently associated with increased mortality in ascitic HCC patients. The ALBI and EZ-ALBI grade can adequately stratify overall survival in both the entire cohort and specifically in HCC patients with ascites.

**Abstract:**

Patients with hepatocellular carcinoma (HCC) often have co-existing ascites, which is a hallmark of liver decompensation. The albumin-bilirubin (ALBI) grade and EZ (easy)-ALBI grade are used to assess liver functional reserve in HCC, but the predictive accuracy of these two models in HCC patients with ascites is unclear. We aimed to determine the prognostic role of ALBI and EZ-ALBI grades in these patients. A total of 4431 HCC patients were prospectively enrolled and retrospectively analyzed. Independent prognostic predictors were identified by the multivariate Cox proportional hazards model. Of all patients, 995 (22.5%) patients had ascites. Grade 1, 2, and 3 ascites were found in 16%, 4%, and 3% of them, respectively. A higher ascites grade was associated with higher ALBI and EZ-ALBI scores and linked with decreased overall survival. In the Cox multivariate analysis, serum bilirubin level > 1.1 mg/dL, creatinine level ≥ 1.2 mg/dL, α-fetoprotein ≥ 20 ng/mL, total tumor volume > 100 cm^3^, vascular invasion, distant metastasis, poor performance status, ALBI grade 2 and 3, EZ-ALBI grade 2 and 3, and non-curative treatments were independently associated with increased mortality (all *p* < 0.05) among HCC patients with ascites. The ALBI and EZ-ALBI grade can adequately stratify overall survival in both the entire cohort and specifically in patients with ascites. Ascites is highly prevalent and independently predict patient survival in HCC. The ALBI and EZ-ALBI grade are feasible markers of liver dysfunction and can stratify long-term survival in HCC patients with ascites.

## 1. Introduction

Hepatocellular carcinoma (HCC) is the most common type of primary liver cancer and the third leading cause of cancer-related mortality globally in 2020 [1]. Chronic hepatitis B and C, alcohol, and non-alcoholic fatty liver disease (NAFLD) are the main risk factors for liver cirrhosis and HCC [2,3]. According to current HCC clinical guidelines, curative treatments such as partial hepatectomy, local ablation therapy, and liver transplantation are recommended for very early or early stage HCC. For intermediate or advanced-stage HCC, transarterial chemoembolization (TACE), systemic therapy, and immunotherapy are usually indicated [4,5,6,7].

HCC typically arises in the background of liver cirrhosis. Ascites, as a hallmark of advanced cirrhosis [8], is a sign of liver decompensation and often coexist in patients with HCC. In addition to the factor of cirrhosis, ascites formation may also result from a large tumor burden [9]. Therefore, ascites is not only an indicator of liver dysfunction but also a signal of tumor aggressiveness.

The Child–Turcotte–Pugh (CTP) score is traditionally used to assess the severity of liver cirrhosis and has been included in most HCC staging systems [6,10]. However, the CTP classification has apparent disadvantages, such as arbitrarily defined cutoff points and interrelated variables. Recently, the albumin-bilirubin (ALBI) score, based solely on serum albumin and bilirubin level, is an objective parameter of liver functional reserve in HCC and has been validated by several independent research groups [11,12,13]. However, the ALBI score has a potential drawback due to the complexity of calculations. Kariyama and colleagues proposed an updated version, the easy (EZ)-ALBI score, which is much easier to calculate and highly correlated with the ALBI score [14]. In addition, the EZ-ALBI score can discriminate different outcomes from early to advanced stage HCC [15,16]. Given so, the predictive accuracy of ALBI and EZ-ALBI score in HCC patients with ascites is unclear. In this study, we aimed to determine the prognostic role of ALBI and EZ-ALBI scores in a large cohort of HCC patients specifically with ascites. 

## 2. Materials and Methods

### 2.1. Patient Characteristics

Between 2001 to 2020, a total of 4431 HCC patients admitted to Taipei Veterans General Hospital were prospectively enrolled and retrospectively analyzed. Their baseline demographics including age, sex, serum biochemistry, tumor burden (tumor size and nodule), vascular invasion, distant metastasis, liver functional reserve, tumor staging, and treatments were recorded at the time of diagnosis. Patients were followed up every 3–6 months until death or drop out from the follow-up program. This study has been approved by the institutional review board (IRB) of Taipei Veterans General Hospital, Taiwan. The study protocol complies with the standards of the Declaration of Helsinki and current ethical guidelines.

### 2.2. Diagnosis and Treatment

The diagnosis of HCC was confirmed by typical image findings according to current guidelines [2,6]. Vascular invasion was defined as radiological evidence of tumor invasion to branch or main portal vein, or inferior vena cava as described previously [17]. Physical status was assessed by using the Eastern Cooperative Oncology Group (ECOG) performance scale [18].

Ascites was defined as free peritoneal fluid identified by ultrasound or computed tomography. Grading of ascites was based on the quantitative criteria by the European Association for the Study of Liver (EASL) guidelines: grade 1 or mild ascites, only detectable by ultrasonography; grade 2 or moderate ascites, denoted by a mild symmetrical abdominal distension; grade 3 or large ascites, indicated by marked abdominal distension [19].

The newly diagnosed patients were discussed at the multidisciplinary board that included hepatologists, oncologists, surgeons, pathologists, and radiologists for diagnosis and treatment strategy. Shared-decision making was completed between patients and physicians. Surgical resection, liver transplantation, and local ablation therapy were collectively denoted as curative treatments; TACE, targeted or systemic therapy were grouped as non-curative treatments. 

### 2.3. Total Tumor Volume (TTV)

TTV was calculated according to the following mathematical equation:

Tumor volume (cm^3^) = 4/3 × 3.14 × (maximum radius of the tumor nodule in cm)^3^

TTV was the sum of the tumor volume of every nodule:

TTV (cm^3^) = tumor volume of (tumor nodule 1 + tumor nodule 2 + ... tumor nodule N) [20].

### 2.4. ALBI and EZ-ALBI Score

ALBI score = (log10 bilirubin (umol/L) × 0.66 – (albumin (g/L) × 0.085) 

The cut-off value of ALBI grade 1/2 and grade 2/3 were ≤−2.60 and >−1.39, respectively [11]. 

EZ-ALBI score = total bilirubin (mg/dL) – (9 × albumin (g/dL))

The cut-off values of EZ-ALBI grade 1/2 and grade 2/3 were ≤−34.4 and >−22.2, respectively [14].

### 2.5. Statistics

The statistical analyses were performed by using IBM SPSS Statistics for Windows, version 25.0 (IBM Corp., Armonk, NY, USA). Continuous variables were analyzed by the Mann–Whitney rank-sum test, and chi-squared test or two-tailed Fisher’s exact test were used to compare categorical variables. The overall survival (OS) was evaluated by the Kaplan–Meier analysis with a log-rank test. Factors that were significant in the univariate survival analysis were entered into the Cox proportional hazards model to determine the independent predictors and the adjusted hazard ratio (HR) and 95% confidence interval (CI). 

## 3. Results

### 3.1. Patient Characteristics

The comparison of baseline characteristics between patients with and without ascites is shown in Table 1. A total of 995 (22.5%) patients had ascites at the time of diagnosis. Patients with ascites were associated with lower serum albumin levels, higher bilirubin levels, higher creatinine levels, lower sodium levels, prolonged prothrombin time, higher platelet count, higher serum α-fetoprotein (AFP) levels, large and multiple tumors, higher rate of vascular invasion, higher CTP, ALBI and EZ-ALBI score, poor performance status, advanced Barcelona Clinic Liver Cancer (BCLC) stage, and a higher rate of receiving non-curative treatments or best supportive care as compared with those without ascites (all *p* < 0.001).

### 3.2. Association between ALBI Score and EZ-ALBI Score for HCC Patients with Ascites

**Figure 1:**
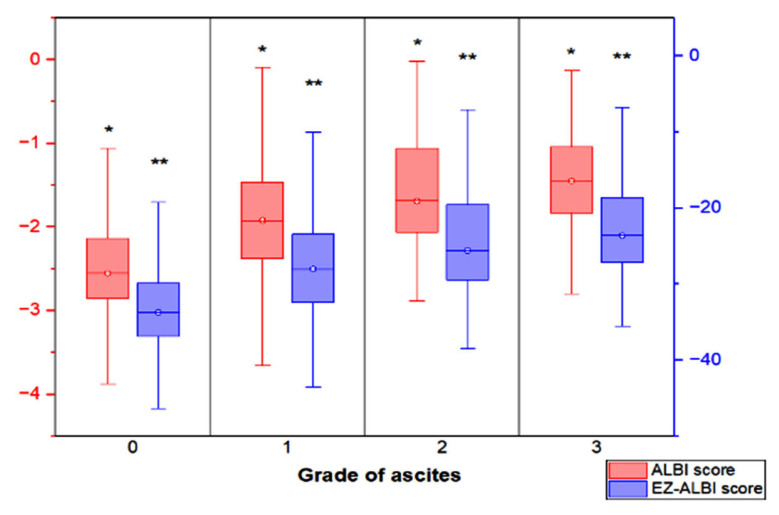
The box plot showing the distribution of ALBI score and EZ-ALBI score in HCC patients with different grades of ascites and without ascites. Patients with higher grade of ascites had both higher ALBI and EZ-ALBI scores (*p* < 0.001). The interquartile range (box), median (horizontal line), and range (vertical lines) values were presented with box-and-whisker plot of the ALBI score and EZ-ALBI score. * *p* < 0.001, ** *p* < 0.001.

The distribution of ALBI and EZ-ALBI scores in patients with different grades of ascites and without ascites is shown in Figure 1. Patients with higher grades of ascites more often had higher ALBI and EZ-ALBI scores (*p* < 0.001).

### 3.3. Survival Analysis in Patients with and without Ascites

**Figure 2:**
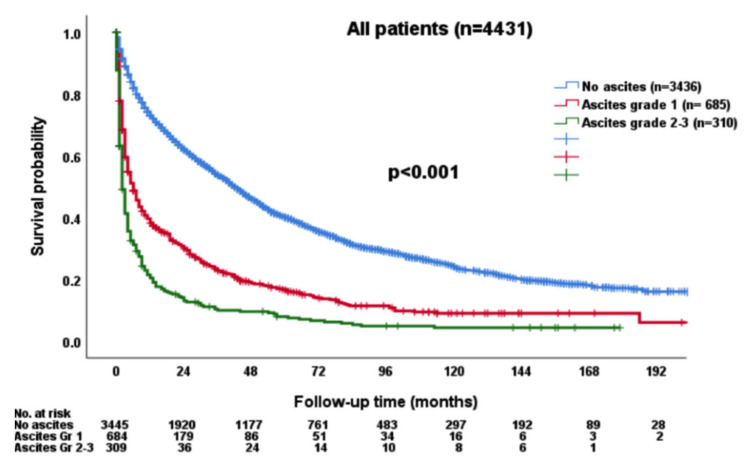
The survival distribution according to the grade of ascites. Patients with higher grades of ascites had worse overall survival compared with patients without ascites or with lower grade ascites (*p* < 0.001).

Patients with ascites grade 2/3 had poor OS compared to those without ascites or lower grade ascites (*p* < 0.001; Figure 2).

### 3.4. Survival Analysis Based on ALBI and EZ-ALBI Grade 

**Figure 3:**
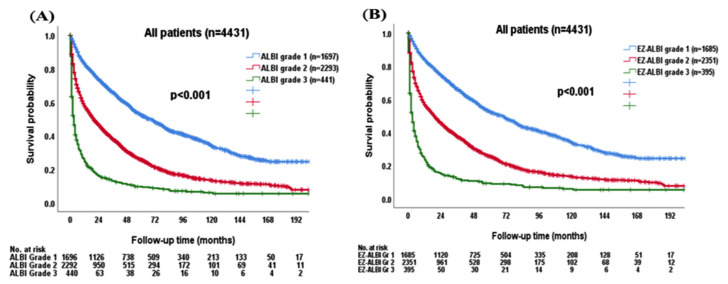
The survival distribution in HCC patients (*n* = 4431) according to different (**A**) ALBI grade and (**B**) EZ-ALBI grade. Patients had worse overall survival in both higher ALBI grade and EZ-ALBI grade (*p* < 0.001).

The survival distribution was further stratified by ALBI and EZ-ALBI grade in the entire cohort. Patients with ALBI grade 2 or 3 had an increased risk of mortality compared with ALBI grade 1 patients (*p* < 0.001; Figure 3A). The median OS was 67 (95% CI, 60.4–73.6) months for ALBI grade 1, 20 (95% CI, 17.7–22.3) months for grade 2, and 3 (95% CI, 2.4–3.6) months for grade 3 patients, respectively. The 1-, 3-, and 5-year OS rates were 83%, 66%, and 52% for ALBI grade 1, 58%, 38%, and 26% for grade 2, and 24%, 13%, and 9% for grade 3 patients, respectively. Patients with EZ-ALBI grade 1 had better OS compared with EZ-ALBI grade 2 and 3 patients (*p* < 0.0001; Figure 3B). The median OS was 69 (95% CI, 62.2–75.8) months for EZ-ALBI grade 1, 19 (95% CI, 16.7–21.3) months for grade 2, and 3 (95% CI, 2.3–3.7) months for grade 3 patients, respectively. The 1-, 3-, and 5-year OS rates were 84%, 66%, and 52% for EZ-ALBI grade 1, 57%, 37%, and 25% for grade 2, and 23%, 12%, and 9% for grade 3 patients, respectively.

### 3.5. Survival Analysis in Patients with and without Ascites Stratified by ALBI Grade 

**Figure 4:**
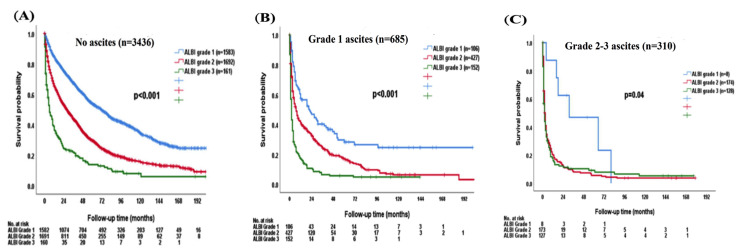
The survival distribution of different ALBI grades according to patients (**A**) without ascites, (**B**) grade 1 ascites, (**C**) grade 2–3 ascites. The ALBI grade can discriminate overall survival in patients without ascites (*p* < 0.001), grade 1 ascites (*p* < 0.001), and grade 2–3 ascites (*p* = 0.04).

For patients without ascites, ALBI grade 2 and 3 patients had decreased OS compared with ALBI grade 1 patients (*p* < 0.001; Figure 4A). The median OS was 72 (95% CI, 65–79) months for ALBI grade 1, 28 (95% CI, 24.9–31.8) months for grade 2, and 6 (95% CI, 3.3–8.7) months for grade 3 patients, respectively. The 1-, 3-, and 5-year OS rates were 85%, 66%, and 54% for ALBI grade 1, 66%, 44%, and 30% for grade 2, and 37%, 21%, and 14% for grade 3 patients, respectively. 

There was significant survival difference stratified by ALBI grade in patients with grade 1 ascites (*p* < 0.001; Figure 4B). The median OS was 67 (95% CI, 60.4–73.6) months for ALBI grade 1, 20 (95% CI, 17.7–22.3) months for grade 2, and 3 (95% CI, 2.4–3.6) months for grade 3. The 1-, 3-, and 5-year OS rates were 85%, 68%, and 54% for ALBI grade 1, 66%, 44%, and 30% for grade 2, and 37%, 22%, and 13% for grade 3 patients with grade 1 ascites, respectively. The ALBI grade 1 patients had better OS compared with ALBI grade 2 or 3 in grade 2/3 ascites patients (*p* = 0.04; Figure 4C). The median OS was 18 (95% CI, 0–63.3) months for ALBI grade 1, 3 (95% CI, 2.4–3.6) months for grade 2, and 2 (95% CI, 1.3–2.7) months for grade 3 patients with grade 2/3 ascites. The 1-, 3-, and 5-year OS rates were 62%, 50%, and 25% for ALBI grade 1, 20%, 9%, and 6% for grade 2, and 18%, 9%, and 7% for grade 3 patients with grade 2/3 ascites, respectively.

### 3.6. Survival Analysis in Patients with and without Ascites Stratified by EZ-ALBI Grade

**Figure 5:**
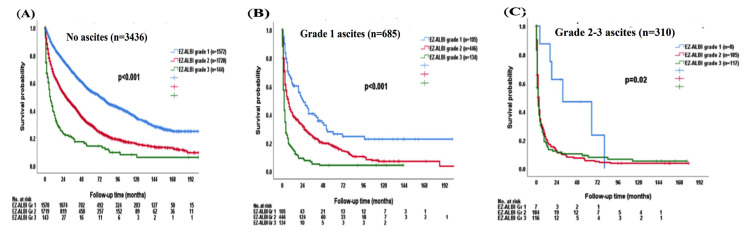
The survival distribution of different EZ-ALBI grades according to patients (**A**) without ascites, (**B**) grade 1 ascites, (**C**) grade 2–3 ascites. The EZ-ALBI grade can discriminate overall survival in patients without ascites (*p* < 0.001), grade 1 ascites (*p* < 0.001), and grade 2–3 ascites (*p* = 0.02).

Consistently, there was a significant survival difference according to different EZ-ALBI grades in patients without ascites (*p* < 0.001; Figure 5A); the median OS was 72 (95% CI, 65–79) months for EZ-ALBI grade 1, 28 (95% CI, 24.8–31.2) months for grade 2, and 6 (95% CI, 3.2–8.8) months for grade 3 patients. The 1-, 3-, and 5-year OS rates were 85%, 68%, and 54% for EZ-ALBI grade 1, 65%, 44%, and 30% for grade 2, and 36%, 20%, and 14% for grade 3 patients without ascites, respectively. Patients with EZ-ALBI grade 1 had better OS compared with EZ-ALBI grade 2 and grade 3 patients with grade 1 ascites (*p* < 0.001; Figure 5B). The median OS was 24 (95% CI, 14.4–33.6) months for EZ-ALBI grade 1, 7 (95% CI, 5.3–8.7) months for grade 2, and 2 (95% CI, 1.4–2.6) months for grade 3 among patients with grade 1 ascites. The 1-, 3-, and 5-year OS was 61%, 39%, and 26% for EZ-ALBI grade 1, 40%, 24%, and 17% for grade 2, and 16%, 5%, and 3% for grade 3 patients with grade 1 ascites, respectively. Patients with EZ-ALBI grade 2 and grade 3 had decreased OS compared with EZ-ALBI grade 1 patients in grade 2/3 ascites (*p* = 0.02; Figure 5C). The median OS was 31 (95% CI, 0–75) months for EZ-ALBI grade 1, 3 (95% CI, 2.4–3.6) months for grade 2, and 2 (95% CI, 1.3–2.7) months for grade 3 patients with grade 2/3 ascites. The 1-, 3-, and 5-year OS were 75%, 47%, and 24% for EZ-ALBI grade 1, 20%, 8%, and 5% for grade 2, and 16%, 10%, and 8% for grade 3 patients with grade 2/3 ascites, respectively.

### 3.7. Multivariate Cox Analysis

In univariate analysis of the entire cohort (*n* = 4431), gender, older age, HBV infection, low platelet count, lower serum albumin level, higher serum bilirubin, creatinine, ALT, and α-fetoprotein (AFP) level, coagulopathy, presence of vascular invasion, distant metastasis, presence of diabetes mellitus, ALBI grade 2 and grade 3, poor performance status, non-curative treatment, tumor size, tumor nodule and total tumor volume, and presence of ascites were associated with decreased survival in the Cox multivariate model 1 (Table 2). The Cox analysis revealed that age > 65 years, serum bilirubin level > 1.1 mg/dL, albumin level < 3.5 g/dL, creatinine ≥ 1.2 mg/dL, AFP ≥ 20 ng/mL, vascular invasion, distant metastasis, ALBI grade 2, ALBI grade 3, performance status 1, performance status 2–4, non-curative treatment, tumor size > 3 cm, multiple tumor nodules, TTV > 100 cm^3^, ascites grade 1 and ascites grade 2–3 were associated with decreased OS (all *p* < 0.01). 

Since ALBI grade and EZ-ALBI grade are highly associated variables, we investigated the role of EZ ALBI to replace ALBI in the Cox model 2 (Table 2). Multivariate analysis showed that age> 65 years, serum bilirubin level > 1.1 mg/dL, albumin level < 3.5 g/dL, creatinine ≥ 1.2 mg/dL, AFP ≥ 20 ng/mL, vascular invasion, distant metastasis, EZ-ALBI grade 2, EZ-ALBI grade 3, performance status 1, performance status 2–4, non-curative treatment, tumor size > 3 cm, multiple tumor nodules, TTV > 100 cm^3^, ascites grade 1 and ascites grade 2–3 were associated with decreased OS (all *p* < 0.01).

### 3.8. Multivariate Cox Analysis in Patients with Ascites

Using a similar approach, the prognostic predictors in 995 HCC patients with ascites were identified. In Cox model 1 (Table 3), serum bilirubin level > 1.1 mg/dL, creatinine ≥ 1.2 mg/dL, AFP ≥ 20 ng/mL, vascular invasion, distant metastasis, ALBI grade 3, performance status 1, performance status 2–4, non-curative treatment, TTV > 100 cm^3^ were associated with decreased OS in HCC patients with ascites (all *p* < 0.001). 

Alternatively, in Cox model 2 which used EZ-ALBI grade (Table 3), serum bilirubin level > 1.1 mg/dL, creatinine ≥ 1.2 mg/dL, AFP ≥ 20 ng/mL, vascular invasion, distant metastasis, EZ-ALBI grade 2, EZ-ALBI grade 3, performance status 1, performance status 2–4, non-curative treatment, TTV > 100 cm^3^ were linked with decreased OS in these patients (all *p* < 0.001).

## 4. Discussion

In this study, ascites was identified in 22.5% of a large cohort of 4431 HCC patients. We show that the presence of ascites in HCC was strongly associated with decreased survival compared with those without ascites. Since ALBI and EZ-ALBI grades are markers of liver dysfunction, we further investigated the prognostic role of ALBI and EZ-ALBI grades in this special patient group. Our data consistently reveal that ALBI and EZ-ALBI grades are feasible prognostic surrogates in HCC patients with different grades of ascites in terms of predicting their long-term survival. 

Ascites is one of the major complications of liver cirrhosis [21]. It is well known that patients with ascites had an increased risk of mortality compared with those without ascites [22]. Consistently, HCC patients with ascites had higher CTP, ALBI, and EZ-ALBI scores in this study. In addition, patients with ascites manifested higher serum creatinine levels and lower serum sodium levels. These findings suggest a strong link between ascites and cirrhosis-associated factors. In HCC patients with ascites, renal insufficiency and hyponatremia were associated with an increased risk of mortality, and our results were consistent with previous studies [23,24]. Alternatively, patients with ascites often had larger tumor burdens, higher rates of vascular invasion or metastasis, and higher AFP levels. The data are in line with the notion that ascites may bear a close relationship with tumor aggressiveness. Notably, ascites can also be found in patients with large tumors without apparent cirrhosis; the main reason may be due to the large tumor burden with vascular invasion which subsequently induced portal hypertension in these patients. Therefore, ascites is not only a result of the poor liver reserve but also could be a surrogate to indicate cancer aggressiveness.

The severity of liver dysfunction plays a crucial role in the management of HCC. CTP score is a commonly used method to evaluate the severity of liver cirrhosis, but it has intrinsic drawbacks due to the inclusion of subjective variables. ALBI and EZ-ALBI scores are alternative markers to indicate liver reserve, and previous reports showed that they can well stratify patient survival in HCC [11,14]. In this study, ALBI and EZ-ALBI grade 1 patients consistently had the highest OS compared with grade 2 and 3 patients in the entire cohort and in patients with different grades of ascites. In the multivariate analysis, patients with ALBI grade 2 or 3 had a 1.3- to 1.7-fold increased risk of mortality compared with ALBI grade 1 patients with ascites. Similarly, EZ-ALBI grade 2 or 3 patients had 1.5- to 1.8-fold increased risk of death compared with EZ-ALBI grade 1 patients with ascites. Taken together, our results confirm that ALBI and EZ-ALBI grades are feasible makers to discriminate long-term outcomes in all HCC patients and in patients specifically with ascites. 

Other findings in this study are also consistent with most previous studies [22,24,25]: elevated serum creatinine level, low serum sodium level, poor performance status, and non-curative treatments were independent predictors of poor survival in HCC patients with ascites. These data indicate that baseline characteristics and treatment modality are tightly linked with the outcome of these patients.

This study has a few limitations. Firstly, our finding is based on a single center in the Asia-Pacific region, and about half of our patients had HBV infection as the predominant etiology of HCC. This feature is quite different from most Western countries and Japan where HCV is the main etiology. Secondly, although the grade of ascites has been defined in the EASL guidelines, the grading of ascites in HCC was mostly based on the clinician’s subjective judgment. In addition, clinical interventions such as paracentesis and diuretics use may also interfere with the objective evaluation for ascites grading.

## 5. Conclusions

Ascites is common and is an independent prognostic predictor of HCC; patients with ascites are at an increased risk of mortality. The ALBI and EZ-ALBI grades are useful makers in predicting the outcome of HCC patients specifically with ascites. Further external validation is required to confirm our findings. 

## Figures and Tables

**Table 1:** Baseline characteristics of hepatocellular carcinoma patients with and without ascites (*n* = 4431).

Variables	Without Ascites (*n* = 3436)	With Ascites (*n* = 995)	*p* Value
Age (years, mean ± SD)	65 ± 13	65 ± 14	0.026
Male/female, *n* (%)	2595/841 (75/25)	757/238 (76/24)	0.719
Etiology of liver disease			
HBV, *n* (%)	1666 (48)	490 (49)	0.781
HCV, *n* (%)	874 (25)	217 (22)	0.017
HBV + HCV, *n* (%)	154 (5)	42 (4)	0.725
Others, *n* (%)	742 (22)	246 (25)	0.037
Laboratory values			
Albumin (g/dL)	3.8 ± 0.6	3.2 ± 0.6	<0.001
Bilirubin (mg/dL)	1.20 ± 2.21	2.67 ± 4.18	<0.001
ALT (IU/L)	67 ± 94	79 ± 138	0.166
Creatinine (mg/dL)	1.11 ± 0.90	1.31 ± 1.24	<0.001
Sodium (mmol/L)	139 ± 4	136 ± 5	<0.001
INR of PT	1.08 ± 0.36	1.21 ± 0.28	<0.001
Platelets (1000 μL/L)	170 ± 89	182 ± 114	<0.001
Serum AFP (ng/mL), median (IQR)	29.5 (6–382)	178.5 (12–6705)	<0.001
Tumor nodules (single/multiple), *n* (%)	2340(68)/1096(32)	559(56)/436(44)	<0.001
Tumor size (cm, mean ± SD)	5.4 ± 4.14	8 ± 5.13	<0.001
Tumor size > 3 cm, *n* (%)	2108 (61)	764 (77)	<0.001
Vascular invasion, *n* (%)	1051 (31)	473 (48)	<0.001
Distant metastasis, *n* (%)	1350 (39)	209 (21)	<0.001
Ascites, *n* (%)			
Grade 1		685 (69)	
Grade 2		164 (17)	
Grade 3		146 (14)	
DM, *n* (%)	891 (26)	304 (31)	0.004
CTP class, A/B/C, *n* (%)	3061/358/17 (89/10/1)	224/592/179 (22/60/18)	<0.001
CTP score, (mean ± SD)	5 ± 1	8 ± 2	<0.001
ALBI score, (mean ± SD)	−2.46 ± 0.56	−1.78 ± 0.67	<0.001
ALBI grade, 1/2/3, *n* (%)	1583/1692/161 (46/49/5)	114/601/280 (11/60/29)	<0.001
EZ-ALBI score, (mean ± SD)	−33 ± 5.9	−26.1 ± 7.8	<0.001
EZ-ALBI grade, 1/2/3, *n* (%)	1572/1720/144 (46/50/4)	113/631/251 (11/64/25)	<0.001
Performance status, 0/1/2/3–4, *n* (%)	2413/569/325/129 (70/17/9/4)	242/280/226/247 (24/28/23/25)	<0.001
BCLC, 0/A/B/C/D, *n* (%)	322/1053/708/1216/137 (10/31/20/35/4)	12/82/49/536/316 (1/9/5/54/31)	<0.001
Treatment, *n* (%)			
Surgical resection	1290 (37)	88 (9)	<0.001
Liver transplantation	12 (1)	17 (2)	<0.001
Percutaneous ablation	650 (19)	103 (10)	<0.001
TACE	958 (28)	236 (24)	0.009
Chemotherapy or targeted therapy	194 (6)	172 (17)	<0.001
Best supportive care	298 (8)	372 (37)	<0.001

Data are shown as *n* (%), mean ± SD, or median (interquartile range). Abbreviations: AFP, α-fetoprotein; ALBI, albumin–bilirubin; ALT, alanine aminotransferase; BCLC, Barcelona Clinic Liver Cancer; CTP, Child–Turcotte–Pugh; DM, diabetes mellitus; EZ-ALBI, easy ALBI; HBV, hepatitis B virus; HCV, hepatitis C virus; INR, international normalized ratio; PT, prothrombin time; TACE, transarterial chemoembolization.

**Table 2:** Univariate and multivariate survival analyses of patients with HCC (*n* = 4431).

				Univariate Analysis			Multivariate Analysis		
Overall Survival	Number	1-Year Survival (%)	3-Year Survival (%)	HR	95% CI	*p*-Value	HR	95% CI	*p*-Value
**Cox model 1**									
Sex (male/female)	3352/1079	62/70	44/51	1.162	1.068–1.264	<0.001			
Age (≤65/65 years)	2174/2257	63/64	49/43	1.268	1.181–1.362	<0.001	1.251	1.161–1.349	<0.001
HBV (negative/positive)	2079/2352	64/64	45/47	0.892	0.831–0.958	0.002			
HCV (negative/positive)	3144/1287	62/69	45/48	0.982	0.909–1.061	0.649			
Platelet (≥150,000/<150,000/μL)	2352/2079	57/72	42/50	0.863	0.808–0.931	<0.001			
Bilirubin level (≤1.1/>1.1 mg/dL)	2905/1526	72/48	54/31	1.834	1.706–1.973	<0.001	1.146	1.054–1.246	0.001
Albumin level (≥3.5/<3.5 g/dL)	2916/1515	75/42	57/25	2.306	2.144–2.480	<0.001	1.193	1.084–1.312	<0.001
Creatinine (<1.2/≥1.2 mg/dL)	3334/1097	67/55	50/35	1.489	1.377–1.610	<0.001	1.241	1.144–1.347	<0.001
ALT (≤40/>40 IU/L)	1971/2460	69/60	52/42	1.304	1.213–1.402	<0.001			
INR of PT (≤1.1/>1.1)	2801/1630	71/52	53/33	1.664	1.548–1.789	<0.001			
Serum AFP (<20/≥20 ng/mL)	1854/2577	80/53	62/34	1.941	1.802–2.091	<0.001	1.504	1.392–1.626	<0.001
Vascular invasion (no/yes)	3380/1051	77/22	57/11	4.079	3.763–4.423	<0.001	1.886	1.713–2.077	<0.001
Distant metastasis (no/yes)	3081/1350	69/22	50/11	3.267	2.930–3.642	<0.001	1.304	1.161–1.465	<0.001
Diabetes mellitus (no/yes)	3236/1195	64/63	47/43	1.121	1.036–1.214	0.005			
ALBI									
Grade 1	1697	83	66		1		1		
Grade 2	2293	58	38	2.105	1.942–2.280	<0.001	1.380	1.252–1.521	<0.001
Grade 3	441	24	12	4.606	4.606–5.191	<0.001	1.669	1.412–1.973	<0.001
Performance status									
0	2655	80	60		1		1		
1	849	51	33	1.871	1.708–2.050	<0.001	1.236	1.123–1.361	<0.001
2–4	927	32	17	3.418	3.134–3.728	<0.001	1.458	1.317–1.615	<0.001
Curative/non-curative treatment	2152/2279	88/41	71/22	3.598	3.337–3.879	<0.001	2.048	1.878–2.323	<0.001
Tumor size (≤3 cm/>3 cm)	1559/2872	86/53	68/34	2.228	2.059–2.410	<0.001	1.225	1.107–1.355	<0.001
Tumor nodules (single/multiple)	2899/1532	69/55	51/36	1.525	1.418–1.639	<0.001	1.127	1.045–1.216	0.002
TTV (≤100/>100 cm^3^)	2623/1808	82/38	62/22	2.689	2.502–2.890	<0.001	1.389	1.260–1.530	<0.001
Ascites									
no ascites	3436	73	54		1		1		
Grade 1	685	38	23	2.324	2.117–2.551	<0.001	1.244	1.125–1.375	<0.001
Grade 2–3	310	20	10	3.831	3.377–4.437	<0.001	2.048	1.878–2.232	<0.001
**Cox model 2**									
Sex (male/female)	3352/1079	62/70	44/51	1.162	1.068–1.264	<0.001	1.094	1.002–1.194	0.044
Age (≤65/65 years)	2174/2257	63/64	49/43	1.268	1.181–1.362	<0.001	1.265	1.173–1.364	<0.001
HBV (negative/positive)	2079/2352	64/64	45/47	0.892	0.831–0.958	0.002			
HCV (negative/positive)	3144/1287	62/69	45/48	0.982	0.909–1.061	0.649			
Platelet (≥150,000/<150,000/μL)	2352/2079	57/72	42/50	0.863	0.808–0.931	<0.001			
Bilirubin level (≤1.1/>1.1 mg/dL)	2905/1526	72/48	54/31	1.834	1.706–1.973	<0.001	1.173	1.082–1.272	<0.001
Albumin level (≥3.5/<3.5 g/dL)	2916/1515	75/42	57/25	2.306	2.144–2.480	<0.001	1.187	1.080–1.305	<0.001
Creatinine (<1.2/≥1.2 mg/dL)	3334/1097	67/55	50/35	1.489	1.377–1.610	<0.001	1.228	1.131–1.334	<0.001
ALT (≤40/>40 IU/L)	1971/2460	69/60	52/42	1.304	1.213–1.402	<0.001	1.077	1.000–1.160	0.049
INR of PT (≤1.1/>1.1)	2801/1630	71/52	53/33	1.664	1.548–1.789	<0.001			
Serum AFP (<20/≥20 ng/mL)	1854/2577	80/53	62/34	1.941	1.802–2.091	<0.001	1.505	1.392–1.628	<0.001
Vascular invasion (no/yes)	3380/1051	77/22	57/11	4.079	3.763–4.423	<0.001	1.893	1.719–2.084	<0.001
Distant metastasis (no/yes)	3081/1350	69/22	50/11	3.267	2.930–3.642	<0.001	1.308	1.165–1.470	<0.001
Diabetes mellitus (no/yes)	3236/1195	64/63	47/43	1.121	1.036–1.214	0.005			
EZALBI									
Grade 1	1685	84	66		1		1		
Grade 2	2351	57	37	2.152	1.987–2.332	<0.001	1.424	1.294–1.566	<0.001
Grade 3	395	23	12	4.769	4.208–5.405	<0.001	1.612	1.367–1.902	<0.001
Performance status									
0	2655	80	60		1		1		
1	849	51	33	1.871	1.708–2.050	<0.001	1.239	1.125–1.364	<0.001
2–4	927	32	17	3.418	3.134–3.728	<0.001	1.471	1.329–1.628	<0.001
Curative/non-curative treatment	2152/2279	88/41	71/22	3.598	3.337–3.879	<0.001	2.039	1.870–2.223	<0.001
Tumor size (≤3 cm/>3 cm)	1559/2872	86/53	68/34	2.228	2.059–2.410	<0.001	1.218	1.100–1.348	<0.001
Tumor nodules (single/multiple)	2899/1532	69/55	51/36	1.525	1.418–1.639	<0.001	1.124	1.042–1.213	0.002
TTV (≤100/>100 cm^3^)	2623/1808	82/38	62/22	2.689	2.502–2.890	<0.001	1.365	1.238–1.504	<0.001
Ascites									
no ascites	3436	73	54		1		1		
Grade 1	685	38	23	2.324	2.117–2.551	<0.001	1.241	1.123–1.372	<0.001
Grade 2–3	310	20	10	3.831	3.377–4.437	<0.001	1.537	1.335–1.769	<0.001

Abbreviations: AFP, α-fetoprotein; ALBI, albumin–bilirubin; ALT, alanine aminotransferase; BCLC, Barcelona Clinic Liver Cancer; CTP, Child–Turcotte–Pugh; DM, diabetes mellitus; EZ-ALBI, easy ALBI; HBV, hepatitis B virus; HCV, hepatitis C virus; INR, international normalized ratio; PT, prothrombin time; TACE, transarterial chemoembolization; TTV, total tumor volume.

**Table 3:** Univariate and multivariate survival analyses of HCC patients with ascites (*n* = 995).

				Univariate Analysis			Multivariate Analysis		
Overall Survival	Number	1-Year Survival (%)	3-Year Survival (%)	HR	95% CI	*p*-Value	HR	95% CI	*p*-Value
**Cox model 1**									
Sex (male/female)	757/238	30/40	17/25	1.229	1.046–1.445	0.11			
Age (≤65/65 years)	553/462	34/31	21/16	1.159	1.012–1.327	0.033			
HBV (negative/positive)	463/532	36/30	20/18	1.110	0.970–1.271	0.130			
HCV (negative/positive)	736/259	30/40	18/21	0.844	0.724–0.985	0.031			
Platelet (≥150,000/<150,000/μL)	529/466	23/44	12/27	0.638	0.556–0.731	<0.001			
Bilirubin level (≤1.1/>1.1 mg/dL)	397/598	45/25	28/12	1.737	1.509–2.000	<0.001	1.384	1.180–1.624	<0.001
Albumin level (≥3.5/<3.5 g/dL)	652/343	44/27	29/13	1.601	1.383–1.853	<0.001			
Creatinine (<1.2/≥1.2 mg/dL)	686/309	35/27	12/22	1.300	1.127–1.501	<0.001	1.178	1.016–1.366	0.03
ALT (≤40/>40 IU/L)	397/598	39/29	24/16	1.331	1.157–1.531	<0.001			
INR of PT (≤1.1/>1.1)	381/614	36/31	21/17	1.191	1.036–1.369	<0.014			
Serum AFP (<20/≥20 ng/mL)	314/681	52/24	34/12	1.894	1.625–2.208	<0.001	1.358	1.155–1.597	<0.001
Vascular invasion (no/yes)	522/473	52/12	33/3	2.894	2.499–3.352	<0.001	1.692	1.430–2.002	<0.001
Distant metastasis (no/yes)	786/209	39/10	23/2	2.038	1.725–2.408	<0.001	1.303	1.097–1.548	0.003
Diabetes mellitus (no/yes)	691/304	31/38	18/20	0.898	0.775–1.041	0.153			
ALBI									
Grade 1	114	59	40	1			1		
Grade 2	601	35	20	1.852	1.457–2.335	<0.001	1.274	0.99–1.641	0.06
Grade 3	280	17	8	2.891	2.236–3.378	<0.001	1.689	1.264–2.257	<0.001
Performance status									
0	242	59	40	1			1		
1	280	38	20	1.695	1.388–2.071	<0.001	1.462	1.194–1.790	<0.001
2–4	473	16	7	2.983	2.480–3.589	<0.001	1.957	1.612–2.375	<0.001
Curative/non-curative treatment	210/785	78/20	57/8	3.370	3.074–4.525	<0.001	2.246	1.814–2.781	<0.001
Tumor size (≤3 cm/>3 cm)	231/764	66/24	42/12	2.357	1.983–2.802	<0.001			
Tumor nodules (single/multiple)	559/436	39/25	22/14	1.345	1.174–1.540	<0.001			
TTV (≤100/>100 cm^3^)	397/598	56/17	35/7	2.388	2.064–2.763	<0.001	1.434	1.215–1.691	<0.001
**Cox model 2**									
Sex (male/female)	757/238	30/40	17/25	1.229	1.046–1.445	0.11			
Age (≤65/65 years)	553/462	34/31	21/16	1.159	1.012–1.327	0.033			
HBV (negative/positive)	463/532	36/30	20/18	1.110	0.970–1.271	0.130			
HCV (negative/positive)	736/259	30/40	18/21	0.844	0.724–0.985	0.031			
Platelet (≥150,000/<150,000/μL)	529/466	23/44	12/27	0.638	0.556–0.731	<0.001			
Bilirubin level (≤1.1/>1.1 mg/dL)	397/598	45/25	28/12	1.737	1.509–2.000	<0.001	1.427	1.226–1.660	<0.001
Albumin level (≥3.5/<3.5 g/dL)	652/343	44/27	29/13	1.601	1.383–1.853	<0.001			
Creatinine (<1.2/≥1.2 mg/dL)	686/309	35/27	12/22	1.300	1.127–1.501	<0.001	1.181	1.019–1.370	0.027
ALT (≤40/>40 IU/L)	397/598	39/29	24/16	1.331	1.157–1.531	<0.001			
INR of PT (≤1.1/>1.1)	381/614	36/31	21/17	1.191	1.036–1.369	<0.014			
Serum AFP (<20/≥20 ng/mL)	314/681	52/24	34/12	1.894	1.625–2.208	<0.001	1.368	1.163–1.609	<0.001
Vascular invasion (no/yes)	522/473	52/12	33/3	2.894	2.499–3.352	<0.001	1.701	1.437–2.014	<0.001
Distant metastasis (no/yes)	786/209	39/10	23/2	2.038	1.725–2.408	<0.001	1.315	1.108–1.561	0.002
Diabetes mellitus (no/yes)	691/304	31/38	18/20	0.898	0.775–1.041	0.153			
EZALBI									
Grade 1	113	63	40	1			1		
Grade 2	631	34	19	1.895	1.487–2.414	<0.003	1.454	1.135–1.862	0.003
Grade 3	251	16	7	2.991	2.299–3.891	<0.001	1.825	1.377–2.417	<0.001
Performance status									
0	242	59	40	1			1		
1	280	38	20	1.695	1.388–2.071	<0.001	1.474	1.204–1.805	<0.001
2–4	473	16	7	2.983	2.480–3.589	<0.001	1.984	1.635–2.407	<0.001
Curative/non-curative treatment	210/785	78/20	57/8	3.370	3.074–4.525	<0.001	2.227	1.799–2.755	<0.001
Tumor size (≤3 cm/>3 cm)	231/764	66/24	42/12	2.357	1.983–2.802	<0.001			
Tumor nodules (single/multiple)	559/436	39/25	22/14	1.345	1.174–1.540	<0.001			
TTV (≤100/>100 cm^3^)	397/598	56/17	35/7	2.388	2.064–2.763	<0.001	1.402	1.189–1.655	<0.001

Abbreviations: AFP, α-fetoprotein; ALBI, albumin–bilirubin; ALT, alanine aminotransferase; BCLC, Barcelona Clinic Liver Cancer; CTP, Child–Turcotte–Pugh; DM, diabetes mellitus; EZ-ALBI, easy ALBI; HBV, hepatitis B virus; HCV, hepatitis C virus; INR, international normalized ratio; PT, prothrombin time; TACE, transarterial chemoembolization; TTV, total tumor volume.

## Data Availability

The data presented in this study are available in this article.
